# In Vitro Investigations of Acetohexamide Binding to Glycated Serum Albumin in the Presence of Fatty Acid

**DOI:** 10.3390/molecules25102340

**Published:** 2020-05-17

**Authors:** Agnieszka Szkudlarek, Michał Wilk, Małgorzata Maciążek-Jurczyk

**Affiliations:** 1 Department of Physical Pharmacy, Faculty of Pharmaceutical Siences in Sosnowiec, Medical University of Silesia in Katowice, 40-001 Katowice, Poland; mmaciazek@sum.edu.pl; 2 Institute of Sport Sciences, Jerzy Kukuczka Academy of Physical Education in Katowice, 40-001 Katowice, Poland; m.wilk@awf.katowice.pl

**Keywords:** fluorescence and UV-Vis spectroscopy, glycation, human serum albumin, saturated and unsaturated fatty acids, acetohexamide

## Abstract

The interaction of drugs with human serum albumin (HSA) is an important element of therapy. Albumin affects the distribution of the drug substance in the body, as well as its pharmacokinetic and pharmacodynamic properties. On the one hand, inflammation and protein glycation, directly associated with many pathological conditions and old age, can cause structural and functional modification of HSA, causing binding disorders. On the other hand, the widespread availability of various dietary supplements that affect the content of fatty acids in the body means that knowledge of the binding activity of transporting proteins, especially in people with chronic diseases, e.g., diabetes, will achieve satisfactory results of the selected therapy. Therefore, the aim of the present study was to evaluate the effect of a mixture of fatty acids (FA) with different saturated and unsaturated acids on the affinity of acetohexamide (AH), a drug with hypoglycaemic activity for glycated albumin, simulating the state of diabetes in the body. Based on fluorescence studies, we can conclude that the presence of both saturated and unsaturated FA disturbs the binding of AH to glycated albumin. Acetohexamide binds more strongly to defatted albumin than to albumin in the presence of fatty acids. The competitive binding of AH and FA to albumin may influence the concentration of free drug fraction and thus its therapeutic effect.

## 1. Introduction

**Scheme 1:**
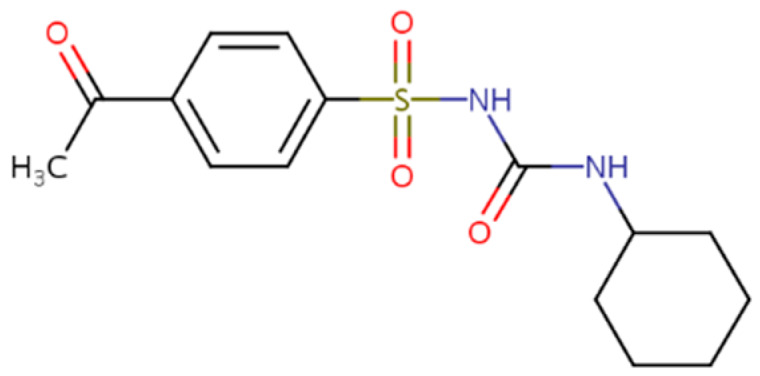
Chemical structure of acetohexamide (AH).

The pace of life, improper eating habits (energy rich, unbalanced diet), and reduction of physical activity to a total minimum has caused an explosion in the number of diabetes all over the world. Diabetes, a civilization related chronic disease, considered a 21st century epidemic, remains a challenge for modern medicine. It is associated with disorders of the mechanisms regulating blood glucose level. Reaserchers argue that the complication and progression of the disease are closely related to the toxic effects of glucose and its metabolites, as one of the pathogenic factors. In patients with the increased blood glucose level, the glycation process intensifies, especially glycation of human serum albumin (HSA) [1]. During the peristent state of hyperglycemia, advanced glycation-end products (AGEs) arise, which are responsible for numerous tissue and organ pathologies [2]. The decrease in glycemia level is crucial to the prevention and inhibition of chronic diabetes (macro- and microvascular) disease progression and complications. This goal is achieved by the change of lifestyle and using anti-diabetic drugs. Acetohexamide (1-((p-acetylphenyl)sulfonyl)-3-cyclohexylurea, AH, Scheme 1) is a first-generation sulfonylurea derivative used to treat type 2 diabetes, especially in people whose blood glucose cannot be controlled by diet. AH is very well absorbed from the gastrointestinal tract and binds to albumin in Sudlow sites I and II [3]. The choice of acetohexamide from many different anti-diabetic drugs was dictated by its physicochemical properties. Its absorbance at wavelengths used to excite the fluorescence of albumin was small enough that allowed high minimize inner filter effect. This is the basic factor limiting the use of the fluorescent technique in drug-protein interactions studies.

**Figure 1:**
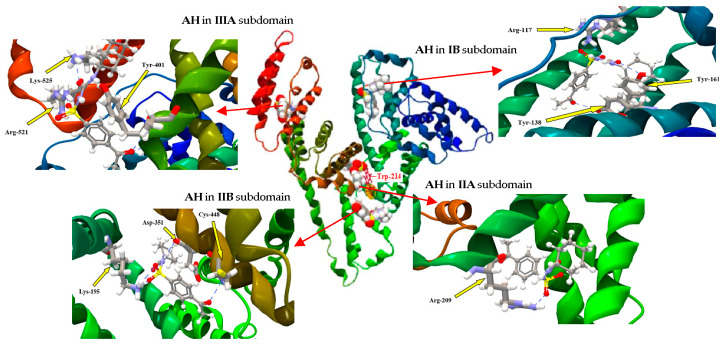
Molecular modeling simulation of acetohexamide (AH) to human serum albumin complexed with glucose (GLC-HSA) (PDB ID: 4IW2).

Figure 1 presents AH binding sites to albumin complexed with glucose (GLC-HSA) within IB, IIB, IIA, and IIIA subdomains and amino acid residues with whom the ligand forms hydrogen bonds.

HSA belongs to the group of soluble proteins most abundant in blood, where its concentration reaches 40–50 g/L. HSA constitutes 55–65% of all proteins present in plasma [4]. Due to the low molecular weight (66.5–66.9 kDa) compared to other plasma globulins, HSA is involved in many physiological processes that include the regulation of osmotic pressure, pH control, and transport to target tissues of many biologically active compounds (e.g., hormones, acids fatty acids, vitamins, and drugs) [5,6]. The transport of ligands, both exogenous and endogenous in a complex with albumin, is manifested by an increase in the solubility of these compounds in serum, a decrease in their toxicity and protection against their oxidation. In the HSA molecule there are two main specific binding sites for exo- and endogenous substances, referred to as site I (located in subdomain IIA) and II (in subdomain IIIA) according to the Sudlow nomenclature [5,7]. Another small drug binding site in HSA is the digitoxin site [8]. Carter et al. described six binding sites for structurally different ligands: two for binding small, heterocyclic or aromatic carboxylic acids (located in IIA and IIIA subdomains), two for binding of long chain fatty acids and small anionic compounds (located in domain I and III in subdomains B) and two sites for the binding of metal ions, subdomain IIIA. The albumin subdomain IA does not have ligand binding properties, despite homology to IIA and IIIA subdomains that bind most drugs [9]. HSA as a carrier protein is the main transporter of saturated and unsaturated fatty acids (FA). In its structure, it has at least seven asymmetric and heterogeneous fatty acid binding sites, which are also located in the vicinity of most drug binding sites, i.e., in IIA (FA-bindig site 7) and IIIA (FA-binding sites 3 and 4) subdomain of macromolecule [7,10,11]. The FA-binding site with the highest affinity is probably in IIIB albumin subdomain (FA-bindig site 5) [12]. Under physiological conditions, the albumin molecule binds from 0.1 to 2 moles of FA. During fasting or extreme exercises and in some diseases, i.e., diabetes or obesity, the [FA]/[HSA] molar ratio may increase to 6:1 or higher [5,13]. Bojko et al., based on fluorescence studies, concluded that albumin forms a complex with two fatty acid molecules at [FA]/[albumin] molar ratio between 1:1 and 8:1. [14]. Many studies show that fatty acids compete for the binding site in the albumin molecule or interact with drugs [15]. The binding affinity between FA and HSA molecules mainly depends on the chemical properties of the particular acid, i.e., the chain length, both the presence and position of double bonds [16]. In polytherapy, ligands can compete with each other for a binding site and displace each other from albumin binding. The drug that forms a complex with the protein is a pharmacologically inactive spare pool, it is not subject to biotransformation and elimination. Only free drug fractions are responsible for therapeutic effect. Binding of a ligand to albumin can change its spatial conformation, which prevents other ligands from binding, or strengthens existing binding. In addition, the binding of the drug to one binding site can change the structure of other binding sites or their number in the albumin molecule [17,18]. The interaction of drugs with HSA is an important element of therapy, because albumin affects the distribution of the drug substance in the body, as well as its pharmacokinetic and pharmacodynamic properties. On the one hand, inflammation, disease processes, or old age can cause structural and functional modification of HSA, causing binding disorders. On the other hand, the widespread availability of various dietary supplements that affect the content of fatty acids in the body, means that knowledge of the binding activity of transporting proteins, especially in people with chronic diseases, e.g., diabetes, will achieve satisfactory results of the selected therapy. This aspect is important during both the determination of drug doses and with respect to the method of their administration, which is particularly important in multi-drug treatments.

Therefore, the purpose of this work is to evaluate the effect of a mixture of fatty acids (FA) with different saturated and unsaturated acids on the affinity of acetohexamide (AH) for glycated albumin, simulating the state of diabetes in the body. Individual fatty acid mixtures corresponded to the FA content in physiological state and in various clinical states, proceeding with an increased concentration of saturated and unsaturated fatty acids.

## 2. Results and Discussion

In the present work, the quenching of albumin fluorophores fluorescence method has been used. This method is a sensitive tool for studying conformational changes in macromolecules caused by low molecular compounds or/and various processes, including glycation. The main goal of the study was to analyze the interaction of acetohexamide (AH), a drug with hypoglycaemic activity and a sulfonylurea derivative of the first generation, with glycated human serum albumin without (af)gHSA and in the presence of fatty acids ((af)gHSA_phys_, (af)gHSA_4S,_ (af)gHSA_8S,_ (af)gHSA_4US_, (af)gHSA_8US_). According to Stryer theory, the basic condition for the energy transfer to the drug molecule without radiations is not more than 10 nm distance between human albumin fluorophores (tryptophanyl residue (Trp-214) and tyrosyl residues (Tyrs)) and the drug chromophore, which results in the quenching of macromolecule fluorescence [19]. A comparison of the course of fluorescence quenching curves at excitation wavelength λ_ex_ = 275 nm and λ_ex_ = 295 nm was aimed in order to demonstrate the participation of tryptophanyl residue (Trp-214) or/and tyrosyl residues (Tyrs) in drug-albumin interaction. The differences in the course of quenching fluorescence curves excited at λ_ex_ = 275 nm and λ_ex_ = 295 nm indicate participation in the interaction of both Trp-214 residue located in subdomain IIA and tyrosyl residues located in IIA (Tyr-263), IB (Tyr-138, Tyr-140, Tyr-148, Tyr-150, Tyr-161), IIB (Tyr-319, Tyr-332, Tyr-334, Tyr-341, Tyr-353, Tyr-370), and IIIA (Tyr-401, Tyr-411, Tyr-452, Tyr-497) subdoamins [20,21]. The mechanism of drug interaction with albumin can be determined on the basis of Stern–Volmer curves. The linear F_0_/F *= f*(C_drug_) relationship indicates a dynamic or static mechanism of macromolecule fluorescence quenching in the environment of subdomains containing amino acid residues that are involved in the formation of the drug–albumin complex. The positive/negative deviation from the straight line of the Stern–Volmer curve indicates the occurrence of both dynamic and static quenching [22]. Estimation of the distance between the drug molecule and excited albumin fluorophore is possible from the Lehrer modified Stern–Volmer curve (F_0_/∆F = *f*(1/[C_drug_])). Based on this dependence, the Stern–Volmer constant (K_SV_) and the fraction of the initial fluorescence of albumin maximally available for the drug (f_a_) are determined. An increase in the distance between the albumin fluorophore and the drug molecule is manifested by a decrease in the K_SV_ constant. The nature of drug binding to albumin, i.e., determination of binding sites specificity in individual classes of macromolecule binding sites, was determined on the basis of binding isotherms ((r = *f*([L_f_])), whose linear course indicates the non-specific binding of the drug to the hydrophobic surface of albumin. The exponential saturation curve, achieving a “plateau”, characterizes a specific binding sites with high affinity and low binding capacity. The mixed nature of the binding, i.e., specific and non-specific, is illustrated by an isotherm with a course between linear and reaching a “plateau” [23]. There are many methods for the calculation of association constant (K_a_) that characterizes the stability of drug-albumin complex, a determination the number of drug molecules associated with one albumin (*n*) molecule at equilibrium, or for prediction the existence of one or more independent classes of binding sites. In the present work, the Klotz (1/r = *f*(1/[L_f_])) and Hill (log[r/(1-r)] = *f*(log[L_f_])) equation were used to determine the value of (K_a_ and *n*) parameters. In addition, in order to determine whether the binding of the drug molecule to albumin influences the increase of its affinity for other macromolecule binding sites, the Hill coefficients (n_H_) were determined from the Hill linear relationship.

### 2.1. The Interaction of Acetohexamide with Human Serum Albumin in the Absence of Fatty Acids (AH-(af)HSA) and the Influence of Physiological Composition of Fatty Acids on AH-(af)HSA Complex (AH-(af)HSAphys)

**Figure 2:**
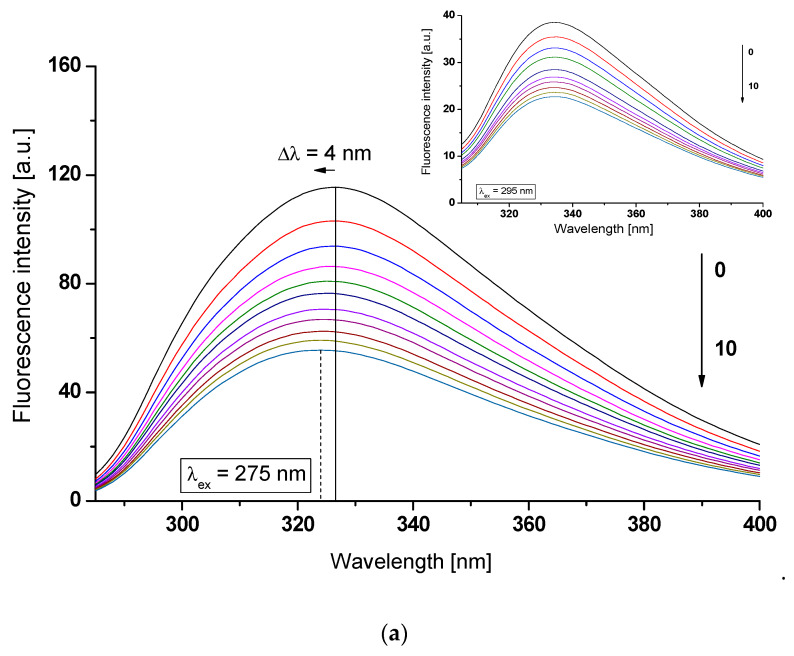

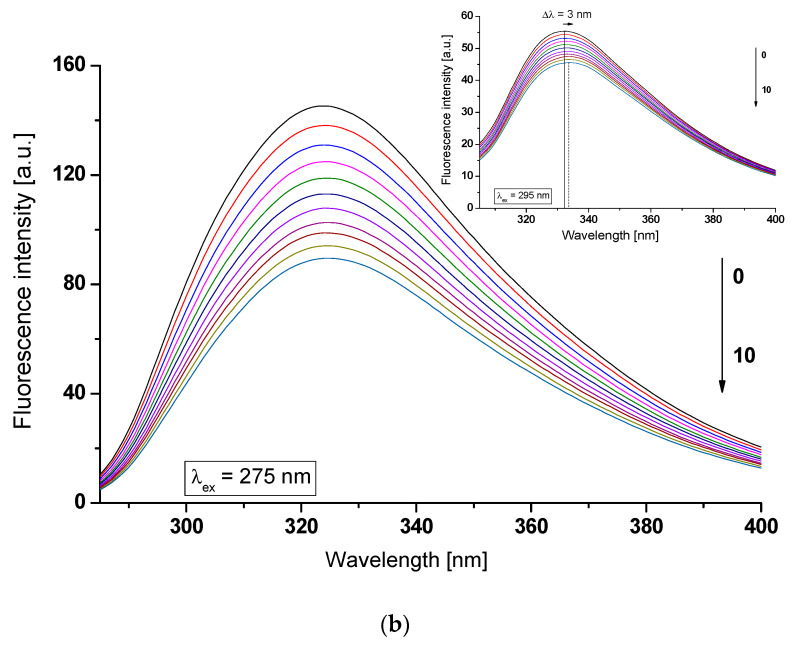
Emission fluorescence spectra of (**a**) (af)gHSA and (**b**) (af)gHSA_phys_ at 5 × 10^−6^ mol∙L^−1^ (0) concentration in the presence of AH at 5 × 10^−6^ mol∙L^−1^ (1) − 5 × 10^−5^ mol∙L^−1^ (10) concentrations, λ_ex_ = 275 nm (in the main view) and λ_ex_ = 295 nm (in the insert); the arrows indicate the shift direction of the maximum fluorescence of (af)gHSA and (af)gHSA_phys_ (Δλ_max_) with the increase of AH concentration; t = 37 °C.

As shown in Figure 2, an increase in acetohexamide (AH) concentration in the AH-(af)gHSA and AH-(af)gHSA_phys_ systems causes a gradual decrease in macromolecule fluorescence intensity. The observed effect may be associated with the quenching fluorescence of excited fluorophores (tryptophanyl residue (Trp-214) and tyrosyl residues (Tyrs)) of albumin (af)gHSA (Figure 2a) and (af)gHSA_phys_ (Figure 2b) by acetohexamide, which was found in no more than 10 nm proximity [19].

In addition, in the AH-(af)gHSA system, after excitation at λ_ex_ = 275 nm, the shift in the albumin fluorescence emission band towards shorter waves (blue-shift) by 4 nm relative to the spectrum of the drug-free albumin (Δλ_max_ = 327–323 nm) (Figure 2a, in the main view) has been observed. The hypsochromic shift indicates a decrease in the polar nature, and thus the formation of a hydrophobic environment around the tryptophanyl residue (Trp-214) and tyrosyl residues (Tyrs) of (af)gHSA due to the interaction of AH with albumin. As in our previous work, the presence of gliclazide (GLZ), a second generation sulfonylurea derivative, also caused a blue-shift of glycated (gHSA_FRC_) spectra in GLZ-gHSA_FRC_ [24]. The blue-shift of maximum albumin fluorescence (Δλ_max_) caused by the presence of AH is associated with a decrease in polarity (increase in hydrophobicity) of albumin fluorophores after binding to the ligand. This indicates the possibility of hydrophobic interactions between the aromatic rings of the acetohexamide molecule and aromatic amino acid rings of the hydrophobic (af)gHSA cavity within IIA (Trp-214, Tyrs) or/and IB, IIB, IIIA (Tyrs) subdomains. For AH-(af)gHSA_phys_ system at excitation wavelength λ_ex_ = 295 nm, a red-shift in fluorescence emission band versus fluorescence of free-drug albumin (af)gHSA_phys_ by 3 nm (Δλ_max_ = 332–335 nm) has been recorded (Figure 2b, in the insert). The shift in the emission fluorescence maximum (af)gHSA_phys_ in the presence of AH towards the long-term direction indicates an increase in polarity and the formation of the hydrophilic environment of albumin fluorophore (Trp-214). Similar observations were noticed by Feng et al. [25] during the fluorescence analysis of theasinesin binding to human serum albumin (HSA). The red-shift of HSA fluorescence spectrum with the increase of ligand concentration the authors explained that tryptophan residue was brought to a more hydrophilic environment in the theasinesin–HSA system, and the structure of the hydrophobic subdomain where a tryptophan was placed was not compact and the segment of polypeptide changed its conformation to a more extended state after the addition of theasinesin. The emission of indole Trp-214 may be blue-shifted if the group is buried within a native protein, and its emission may shift to longer wavelengths (red-shift) when protein is unfolded [20].

**Figure 3:**
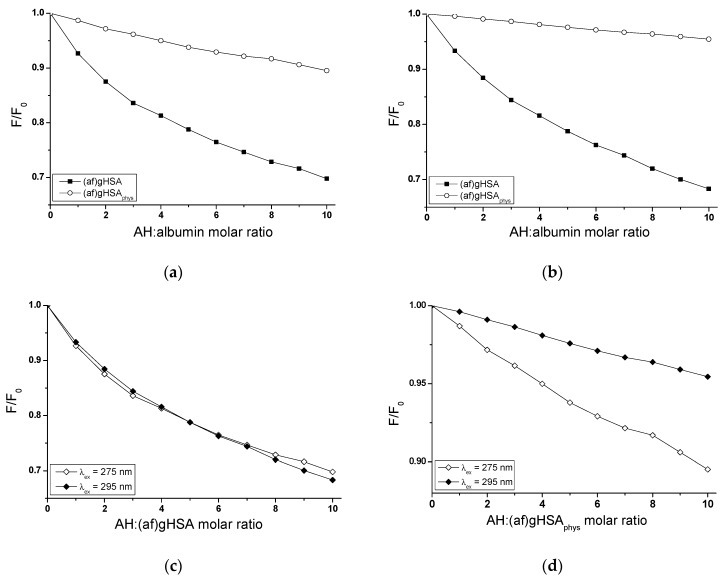
Fluorescence quenching of (af)gHSA and (af)gHSA_phys_ (5 × 10^−6^ mol∙L^−1^) complexed with AH (5 × 10^−6^ mol∙L^−1^ – 5 × 10^−5^ mol∙L^−1^) (**a**) λ_ex_ = 275 nm, (**b**) λ_ex_ = 295 nm and fluorescence quenching of (**c**) AH-(af)gHSA and (**d**) AH-(af)gHSA_phys_ system for λ_ex_ = 275 nm and λ_ex_ = 295 nm; the error bars are smaller than the symbols.

Based on the obtained data from the emission fluorescence spectra, the quenching curves of (af)gHSA and (af)gHSA_phys_ (5 × 10^−6^ mol∙L^−1^) fluorescence in the presence of AH (5 × 10^−6^ mol∙L^−1^ – 5 × 10^−5^ mol∙L^−1^), λ_ex_ = 275 nm and λ_ex_ = 295 nm, have been drawn (Figure 3).

The course of albumin fluorescence quenching curves illustrates the reduction in fluorescence intensity of human serum albumin without (af)gHSA and with (af)gHSA_phys_ fatty acids with the increase of acetohexamide concentration in AH-albumin system (Figure 3a–d). The presence of fatty acids at physiological concentration affects the ability of acetohexsamide to quench albumin fluorescence. For (af)gHSA a stronger fluorescence quenching than for (af)gHSA_phys_, at both excitation wavelengths λ_ex_ = 275 nm (Figure 3a) and λ_ex_ = 295 nm (Figure 3b), has been observed. This phenomenon indicates a greater ability of acetohexamide to absorb energy from defatted albumin than in the presence of fatty acids, that change macromolecule. For ligand: albumin molar ratio 10:1, the quenching of the internal (af)gHSA and (af)gHSA_phys_ fluorescence by AH equals to 30.20% and 10.50%, respectively (λ_ex_ = 275 nm), while λ_ex_ = 295 nm equals to 31.70% and 4.60%, respectively. The comparison of fluorescence quenching curves of (af)gHSA and (af)gHSA_phys_, in the presence of acetohexamide at the excitation wavelength λ_ex_ = 275 nm and λ_ex_ = 295 nm, allowed to indicate the fluorophores (Trp-214 and/or Tyrs) involved in the interaction with AH. Nearly identical course of albumin fluorescence quenching curves in AH-(af)gHSA (Figure 3c) system, at λ_ex_ = 275 nm and λ_ex_ = 295 nm (4.60% difference in quenching of the intrinsic albumin fluorescence), indicates the contribution of a Trp-214 residue (located in IIA subdomain of albumin) or its environment and a negligible contribution of Tyrs residues in the interaction of AH with (af)gHSA in the environment of binding site. A significant difference (56.60%) in quenching of (af)gHSA_phys_ fluorescence registered for λ_ex_ = 275 nm and 295 nm (Figure 3d) indicates the participation in AH interaction with (af)gHSA_phys_ not only Trp-214, but also Tyrs residues. A stronger fluorescence quenching in AH-(af)gHSA_phys_ system at excitation wavelength λ_ex_ = 275 nm than 295 nm may indicate a much easier access of AH to 17 tyrosyl residues (Tyr-30, -84, -138, -140, - 148, -150, -161, -263, -319, -332, -334, -341, -353, -370, -401, -411, -497) located in IB, IIB, IIA, and IIIA [26] subdomains than to the tryptophanyl residue. It is noteworthy that the fluorescence quenching technique is not sufficient to indicate which Tyrs moieties are involved in AH binding. Literature data indicate that in human albumin structure there are two main specific binding sites for endo- and exogenous substances such as acetohexamide. These binding sites defined by Sudlow et al. as site I (in subdomain IIA, where Trp-214, Tyr-263 and His-240 are located) and site II (in subdomain IIIA, where Tyr- 401, Arg-410 and Tyr-411 are located) [5,7].

**Figure 4:**
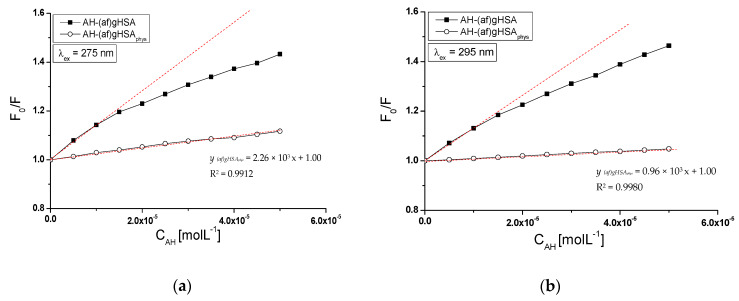
The Stern-Volmer curves for AH-(af)gHSA and AH-(af)gHSA_phys_, (**a**) λ_ex_ = 275 nm, (**b**) λ_ex_ = 295 nm; the error bars are smaller than the symbols.

Based on the data obtained from (af)gHSA and (af)gHSA_phys_ in the presence of AH, the Stern–Volmer curves have been plotted, λ_ex_ = 275 nm (Figure 4a) and λ_ex_ = 295 nm (Figure 4b). The dashed lines indicate a model rectilinear course of Stern–Volmer dependence (F_0_/F = *f*([C_AH_]).

Stern–Volmer curves plotted for glycated, defatted serum albumin in the presence of acetohexamide (AH-(af)gHSA system) show a different course from the curves plotted for AH-(af)gHSA_phys_ system both at excitation λ_ex_ = 275 nm (Figure 4a), and λ_ex_ = 295 nm (Figure 4b). A stronger fluorescence quenching (F_0_/F) at whole range of acetohexamide concentrations have been occurred for albumin (af)gHSA compared to the F_0_/F value obtained for glycated, fatted albumin (af)gHSA_phys_. Negative deviation from the rectilinear relationship F_0_/F = *f*[C_AH_] for AH-(af)gHSA system at λ_ex_ = 275 nm (Figure 4a) and λ_ex_ = 295 nm (Figure 4b), indicates dynamic (collision) and static (formation of a stable ligand-albumin complex in the ground state) quenching fluorescence of glycated, defatted albumin by acetohexamide. Literature data indicate that the negative deviation from the rectilinear course of the Stern–Volmer curve is explained by occuring initially more easily available binding sites for the drug, and after saturating them, those more difficult to access in the macromolecule [27,28].

The rectilinear course of Stern–Volmer plots (F_0_/F = *f*[C_AH_] relationship) obtained for the AH-(af)gHSA_phys_ system may indicate dynamic or static fluorescence quenching mechanism. When the dynamic mechanism takes place, there is a collision of the acetohexamide molecule with albumin (af)gHSA_phys_ fluorophores in excitation state. The static mechanism leads to a decrease in the intensity of emitted fluorescence when acetohexamide binds to the fluorophore molecule in the non-excited state, reducing the population of excited fluorophores [27].

From the F_0_/F = *f*[C_AH_] relationship for a system with a linear course of the Stern–Volmer curve (AH-(af)gHSA_phys_ system), the Stern–Volmer constants K_SV_, the biomolecular quenching rate constants k_q_ (k_q_ = K_SV_/τ_0_) and maximum available fluorescence fraction of all albumin f_a_ fluorophores were determined. Quenching parameters (K_SV_, k_q_ = K_SV_/τ_0_) and f_a_ for a system with non-linear Stern–Volmer relationship (AH-(af)gHSA system) were determined from F_0_/∆F = *f*(1/[C_AH_]) relationship represented by Stern–Volmer equation modified by Lehrer [29]. The obtained results are given in Table 1.

Higher values of K_SV_ and k_q_ constant obtained for the AH-(af)gHSA system compared to K_SV_ and k_q_ obtained for AH-(af)gHSA_phys_ system (Table 1) indicate the location of acetohexamide molecules closer to the fluorophores of glycated, defatted albumin (af)gHSA than fluorophores of fatted by fatty acid physiological mixture albumin ((af)gHSA_phys_). Exciting albumin (af)gHSA and (af)gHSA_phys_ at wavelength λ_ex_ = 275 nm, fluorescence emission was observed not only from the tryptophanyl residue, but also from tyrosyls residues and higher values of K_SV_ constants were obtained than at λ_ex_ = 295 nm excitation wavelength. This demonstrates the contribution of (af)gHSA and (af)gHSA_phys_ tyrosyl residues in AH-albumin interaction. The IIA human serum albumin subdomain contains one tryptophanyl residue (Trp-214) and one tyrosyl residue (Tyr-263), which would indicate that in acetohexamide binding to albumin (af)gHSA and (af)gHSA_phys_ other subdomains, where tyrosyl residues are located (IB, IIB and IIIA subdomains), are also involved. Our observations are consistent with the results obtained by Joseph et al. [3] who, using the high-performance affinity chromatography (HPAC) technique, indicated the IIA and IIIA subdomains, i.e., the I and II binding sites, respectively, as the main binding site of acetohexamide in the glycated human structure serum albumin. The order of fluorescence quenching rate constants k_q_ (10^12^ or 10^11^) determined for AH-(af)gHSA and AH-(af)gHSA_phys_ system clearly indicates a static fluorescence quenching mechanism in the acetohexamide-albumin system (Table 1). According to Lakowicz, when the maximum value of k_q_ constant in the aqueous solution is 1 × 10^10^ [M^−1^s^−1^], the dynamic fluorescence quenching mechanism occurs [20]. Since the values of K_SV_ constant and f_a_ fraction are inversely proportional, the decrease in K_SV_ is accompanied by an increase in f_a_. The presence of fatty acids reduces the strength of AH-(af)gHSA_phys_ fluorophores interaction with the increase the availability (f_a_) of AH to tryptophanyl and tyrosyl (af)gHSA_phys_ residues.

**Figure 5:**
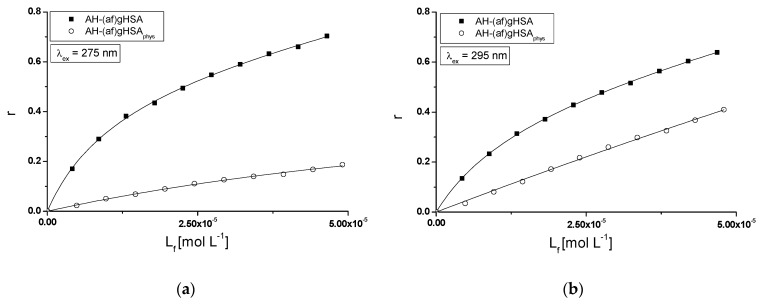
Binding isotherms of (af)gHSA and (af)gHSA_phys_ at 5 × 10^−6^ mol∙L^−1^ concentration with AH at 5 × 10^−6^ − 5 × 10^−5^ mol∙L^−1^, (**a**) λ_ex_ = 275 nm, (**b**) λ_ex_ = 295 nm; the error bars are smaller than the symbols.

To determine the nature of the interaction of acetohexamide with (af)gHSA and (af)gHSA_phys_, saturation curves (binding isotherms) were plotted in the AH-(af)gHSA and AH-(af)gHSA_phys_ systems, λ_ex_ = 275 nm (Figure 5a) and λ_ex_ = 295 nm (Figure 5b).

The shape of the binding isotherms (r = *f*([L_f_]) obtained for (af)gHSA albumin indicates the mixed (specific and non-specific) nature of the interaction, which means that acetohexamide binds not only to its specific binding sites in defatted albumin molecule, but also non-specifically interacts with the hydrophobic fragments of its surface [23]. However, the shape of the binding isotherms for albumin saturated with fatty acids indicates the occurrence of only the non-specific nature of acetohexamide binding to (af)gHSA_phys_ (Figure 5a,b). Specific binding is characterized by high affinity and low binding capacity, while non-specific binding with low affinity and unlimited drug binding capacity [23].

Since acetohexamide saturates the binding sites of defatted ((af)gHSA) and fatted ((af)gHSA_phys_) albumin, using the Klotz (the independent variable is the inverse of the concentration of free ligand fraction, 1/r = *f*(1/[L_f_])) and Hill (the independent variable is the logarithm of ligand free fraction concentration, log[r/(1-r)] = *f*(log[L_f_])) equations, the association constants K_a_, which determine the stability of formed drug-albumin complex, were determined. The number of acetohexamide molecules forming a complex with one molecule of both (af)gHSA and (af)gHSA_phys_ at equilibrium state (*n*) for a specific class of binding sites was also obtained. Moreover, Hill interaction factors (n_H_) were determined to investigate the possible cooperation of acetohexamide binding to the macromolecule. The data are presented in Table 2.

From the straight line of Klotz eguation for AH-(af)gHSA, AH-(af)gHSA_phys_ systems and Hill equation for AH-(af)gHSA, AH-(af)gHSA_phys_ systems, the binding of AH to macromolecules within one class of binding sites has been obtained. For AH-(af)gHSA complex, the association constants K_a_ are more than 10-fold (for λ_ex_ = 275 nm) and more than 5-fold (for λ_ex_ = 295 nm) higher than the constants K_a_ values obtained for AH-(af)gHSA_phys_ complex (Table 2), which indicates that AH has higher affinity for (af)gHSA than for (af)gHSA_phys_ albumin binding sites. This phenomenon is probably caused by the reduction of AH-albumin complex stability in the presence of fatty acids, when both tryptophanyl residue (Trp-214) and tyrosyl residues (Tyrs) have been excited (λ_ex_ = 275 nm). The same results have been received in our other study (data not published). We have concluded that tolbutamide (TB), a hypoglycaemic drug, binds more easily to defatted albumin than in the presence of fatty acid. In the treatment of TB high fat diet increased the therapeutic effect of the drug. The number of binding sites *n* close to unity indicates the existence of one specific AH binding site in the (af)gHSA molecule. Based on n_H_ value (n_H_ ≈ 1) lack of cooperativity in binding of AH to (af)gHSA has been observed (λ_ex_ = 275 nm and λ_ex_ = 295 nm) (Table 2). For AH-(af)gHSA_phys_ complex at λ_ex_ = 295 nm, a Hill coefficient value higher than 1 (n_H_ > 1) points to a positive co-operability phenomenon, which probably means that binding of AH in one place increases the affinity of the drug/drugs for other macromolecule binding sites. The occurrence of cooperative binding results from the fact that under physiological conditions, fatty acid molecules (FA4 and FA5) bind to IIIA and IIIB subdomains [31], thus affecting the albumin conformation, i.e., changes the affinity of a macromolecule for acetohexamide. These observations complement the results discussed earlier that the presence of fatty acids induces changes in the structure of the macromolecule. Under physiological conditions, human serum albumin binds a maximum of two fatty acid moles (FA). The formation of an albumin complex with two FA moles is possible when their molar FA:albumin ratio is within 1:1 and 1:8 [31]. Therefore, in order to assess the binding of AH to glycated human serum albumin in various clinical states, proceeding with increased concentration of saturated (FA_S_) and unsaturated (FA_US_) fatty acids, in the next part of the study albumin with four and eight times more amount of saturated ((af)gHSA_4S_, (af)gHSA_8S_) and unsaturated ((af)gHSA_4US_, (af)gHSA_8US_) fatty acids compared to physiological values have been analyzed.

### 2.2. The Interaction of Acetohexamide with Human Serum Albumin at the Increased Concentration of Saturated ((af)gHSA_4S_, (af)gHSA_8S_) and Unsaturated ((af)gHSA_4US_, (af)gHSA_8US_) Fatty Acids

In the next part of the study, emission fluorescence spectra of glycated albumin at 5 × 10^−6^ mol∙L^−1^ concentration containing four ((af)gHSA_4S_) (4.1 × 10^−5^ mol∙L^−1^) and eight times ((af)gHSA_8S_) (6.9 × 10^−5^ mol∙L^−1^) more saturated fatty acids (FA_S_) and four ((af)gHSA_4US_) (5.9 × 10^−5^ mol∙L^−1^) and eight times ((af)gHSA_8US_) (1.11 × 10^−4^ mol∙L^−1^) more unsaturated (FA_US_) fatty acids in relation to the physiological value (2 × 10^−5^ mol∙L^−1^) have been obtained. Fluorescence of macromolecules excited at λ_ex_ = 275 nm and λ_ex_ = 295 nm decreases with the increase of acetohexamide (AH) concentration (data not shown). Lack of shift in the fluorescence emission band of albumin relative to the albumin spectrum in the presence of AH (Δλ_max_) may indicate the invariability of the hydrophobic/hydrophilic properties of AH binding site (near the Trp-214 residue in subdomain IIA and Tyrs residues in subdomain IB, IIB, IIA, and IIIA of albumins).

**Figure 6:**
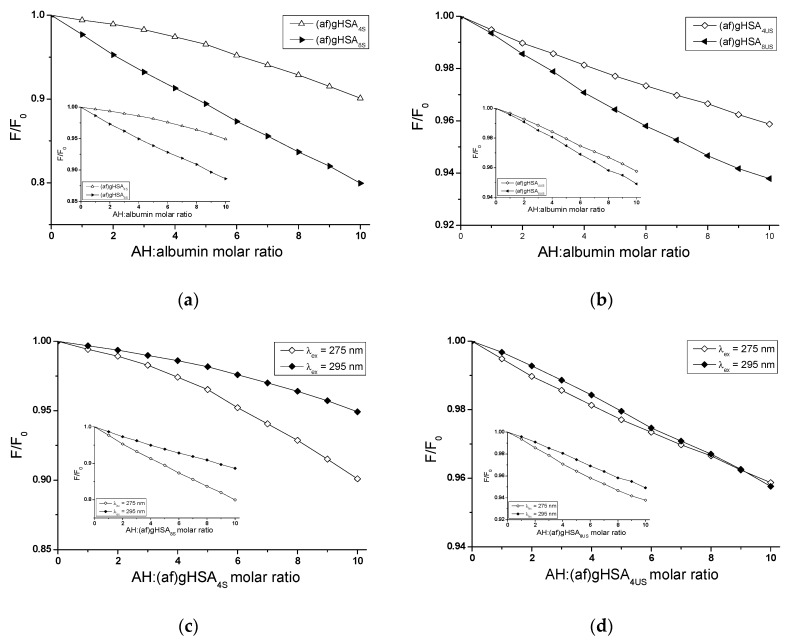
Fluorescence quenching of (**a**) AH-(af)gHSA_4S_ and AH-(af)gHSA_8S_, (**b**) AH-(af)gHSA_4US_ and AH-(af)gHSA_8US_ system for λ_ex_ = 275 nm (in the main view) and λ_ex_ = 295 nm (in the insert); (**c**) AH-(af)gHSA_4S_ (in the main view), AH-(af)gHSA_8S_ (in the insert) and (**d**) AH-(af)gHSA_4US_ (in the main view), AH-(af)gHSA_8US_ (in the insert) system for λ_ex_ = 275 nm and λ_ex_ = 295 nm; the error bars are smaller than the symbols.

As it was previously mentioned, after excitation of albumin at excitation wavelength λ_ex_ = 295 nm, the observed emission of fluorescence comes almost exclusively from the tryptophan residue, while for λ_ex_ = 275 nm from both the tryptophan residue and tyrosyl residues. Differences in quenching of albumin fluorescence at both excitation wavelengths λ_ex_ = 275 nm and λ_ex_ = 295 nm, in AH-(af)gHSA_4S_ (Figure 6c, in the main view), AH-(af)gHSA_8S_ (Figure 6c, in the insert) and AH-(af)gHSA_8US_ (Figure 6d, in the insert) systems, indicate the simultaneously participation of Trp-214 and Tyrs residues in the interaction of AH with albumin at appropriate binding site. The lack of differences in albumin fluorescence quenching curves in AH-(af)gHSA_4US_ system (Figure 6d, in the main view) after excitation at both wavelengths indicates the participation mainly Trp-214 residue.

For albumin with two times more saturated ((af)gHSA_8S_) and unsaturated ((af)gHSA_8US_) fatty acids, a stronger fluorescence quenching with increasing AH concentration has been recorded than for albumin (af)gHSA_4S_ and (af)gHSA_4US_ both after excitation λ_ex_ = 275 nm (Figure 6a,b, in the main view) and λ_ex_ = 295 nm (Figure 6a,b, in the insert). This demonstrates the greater ability of AH to absorb energy from excited fluorophores, as a result of conformational changes caused by a higher content of fatty acids in the structure of albumin.

In addition, for each concentration of AH in the drug-albumin system, the degree of albumin fluorescence quenching is greater after excitation at λ_ex_ = 275 nm than at λ_ex_ = 295 nm, which would indicate a significant parfticipation of tyrosyl residues in the interaction of AH with (af)gHSA_4S_, (af)gHSA_8S_, (af)gHSA_4US_ and (af)gHSA_8US_. Tyrosyl residues in position 401 (Tyr-401) and 411 (Tyr-411) located in IIIA subdomain of macromolecule play a major role in drugs binding [20,32]. The observed significant contribution of Tyrs residues in the interaction of AH with albumin indicates the IIIA subdomain as the likely acetohexamide binding site.

The analysis of Stern–Volmer plots (F_0_/F = *f*[C_AH_]) showed a straight line for AH-(af), AH-(af)gHSA_4US,_ and AH-(af)gHSA_8US_ systems, indicating a collision or static mechanism of albumin quenching fluorescence by AH. However, for AH-(af)gHSA_4S_ system, F_0_/F = *f*[C_AH_] relationship showed a non-linear course (slight deviation in the direction of OY), which would indicate the simultaneous occurrence of dynamic and static fluorescence quenching mechanism, a similar effect that we observed in our previous work [33] concerning the theophylline (Th)–human albumin system study. Quenching parameters (K_SV_, k_q_ = K_SV_/τ_0_) and the fraction of maximum available fluorescence of albumin f_a_, for AH-(af)gHSA_8S_, AH-(af)gHSA_4US_ and AH-(af)gHSA_8US_ were determined from F_0_/F = *f*[C_AH_] relationship, while for AH-(af)gHSA_4S_ from F_0_/∆F = *f*(1/[C_AH_] relationship (Table 3).

An order of quenching constant k_q_ equals to 10^11^ clearly indicates the presence of static fluorescence quenching mechanism of albumin (af)gHSA_8S_, (af)gHSA_4US_ and (af)gHSA_8US_ by AH. A 2-fold increase in saturated (AH-(af)gHSA_8S_ system) and unsaturated (AH-(af)gHSA_8US_ system) fatty acids in albumin (af)gHSA_4S_ and (af)gHSA_4US_, respectively, caused about two-fold (λ_ex_ = 275 nm) and three-fold (λ_ex_ = 295 nm) decrease and about two-fold (λ_ex_ = 275 nm) and one-fold (λ_ex_ = 295 nm) increase in the quenching parameters (K_SV_ and k_q_). This may suggest that in the presence of an increased amount of saturated fatty acids, acetohexamide binds at a considerable distance from the albumin fluorophores, which hinders the transfer of energy between them. However, a greater amount of unsaturated fatty acids facilitates energy transfer in the AH-albumin system (Table 3). In addition, it can be seen that the availability of albumin (af)gHSA_4S_ fluorophores (Trp-214 residues, Tyrs residues) for individual AH binding sites is significantly hindered.

The course of binding isotherms, which determine the binding specificity of ligand to albumin, for AH-(af)gHSA_4S_, AH-(af)gHSA_8S_ and AH-(af)gHSA_4US_, AH-(af)gHSA_8US_ systems is linear. The straight-line relationship of r = *f*([L_f_]) Taira and Terada [23] explained by the non-specific interaction of ligand with the hydrophobic fragments of macromolecule surfaces. However, the association constants K_a_ determined from the Klotz relationship for the complexes AH-(af)gHSA_4S_, AH-(af)gHSA_8S_, AH-(af)gHSA_4US_ and AH-(af)gHSA_8US_ also prove the specific nature of acetohexamide binding within the molecule (Table 4). On the other hand, the non-linear course of Hill dependence obtained for the AH-(af)gHSA_4S_ complex, confirms the interaction of AH mainly with albumin surface fragments. Straight line Klotz plots for AH-(af)gHSA_4S_, AH-(af)gHSA_8S_, AH-(af)gHSA_4US_, AH-(af)gHSA_8US_ and Hill plots for AH-(af)gHSA_8S_, AH-(af)gHSA_4US_ and AH-(af)gHSA_8US_ also indicate the presence of one independent class of AH binding sites in the albumin structure (or one binding site). Binding parameters (K_a_, *n*) and Hill n_H_ coefficient (interaction factor) has been summarized in Table 4.

For AH-albumin complex with two-times the amount of saturated ((af)gHSA_8S_) and unsaturated ((af)gHSA_8US_) fatty acids, the K_a_ constants are smaller than those obtained for the AH- AH-(af)gHSA_4S_ and AH-(af)gHSA_4US_ for λ_ex_ = 275 nm and 295 nm (Table 4). This means that a higher concentration of both saturated and unsaturated fatty acids in glycated albumin reduces the stability of the complex formed with acetohexamide. For AH-(af)gHSA_4S_, AH-(af)gHSA_8S_ and AH-(af)gHSA_4US_, AH-(af)gHSA_8US_ complexes, on average one ligand molecule binds to one albumin molecule (*n* ≈ 1). In contrast, Hill interaction coefficient n_H_ equals to unity (n_H_ ≈ 1) and indicates a lack of cooperativity in the binding of AH to albumin (af)gHSA_8S_, (af)gHSA_4US_ and (af)gHSA_8US_ in the vicinity of Tyrs residues. The same value of n_H_ we received in our previous work [33] when we determinated the Hill interaction coefficient for theophylline (Th)-albumin complex with 8-fold higher saturated fatty acid amount compared to physiological value (Th-dHSA-FA_8S_). n_H_ value greater than unity (n_H_ > 1) obtained for the complexes AH-(af)gHSA_4US_ and AH-(af)gHSA_8US_ excited at λ_ex_ = 295 nm indicates the phenomenon of positive cooperativity, which probably means that the binding of AH in one place (near Trp-214 residue) increases the affinity of the drug/drugs for the rest macromolecule binding sites.

**Figure 7:**
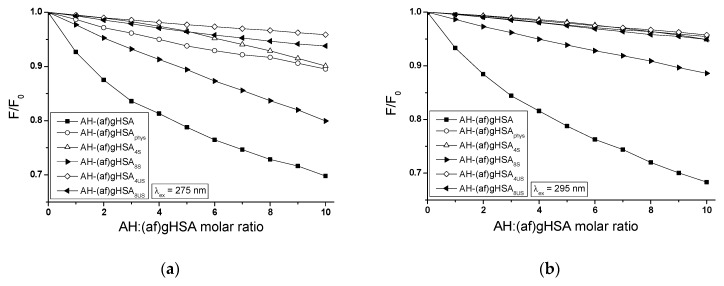
Fluorescence quenching of (af)gHSA, (af)gHSA_phys_, (af)gHSA_4S_, (af)gHSA_8S_, (af)gHSA_4US_, (af)gHSA_8US_ (5 × 10^−6^ mol∙L^−1^) complexed with AH (5 × 10^−6^ mol∙L^−1^ – 5 × 10^−5^ mol∙L^−1^) (**a**) λ_ex_ = 275 nm, (**b**) λ_ex_ = 295 nm; the error bars are smaller than the symbols.

The presence of both saturated and unsaturated fatty acids affects the quenching of glycated, defatted albumin ((af)gHSA) fluorescence by acetohexamide (AH). For albumin (af)gHSA, fluorescence is quenched more strongly with the increase of drug concentration (5 × 10^−6^ mol∙L^−1^ – 5 × 10^−5^ mol∙L^−1^) than for albumin containing four times ((af)gHSA_4S_) (4.1 × 10^−5^ mol∙L^−1^) and eight times ((af)gHSA_8S_) (6.9 × 10^−5^ mol∙L^−1^) more saturated fatty acids (FA_S_) and four times ((af)gHSA_4US_) (5.9 × 10^−5^ mol∙L^−1^) and eight times ((af)gHSA_8US_) (1.11 × 10^−4^ mol∙L^−1^) higher unsaturated (FA_US_) fatty acids in relation to the physiological value (2 × 10^−5^ mol∙L^−1^) (Figure 7).

The largest differences in quenching of internal fluorescence relative to the control system (AH-(af)gHSA) were noted for the AH-(af)gHSA_4US_ system at λ_ex_ = 275 nm (by 26.08%) and λ_ex_ = 295 nm (by 27.44%), while the smallest differences for AH-(af)gHSA_8S_ at λ_ex_ = 275 nm (by 10.15%) and at λ_ex_ = 295 nm (by 20.29%) have been recorded. Stronger fluorescence quenching in AH-(af)gHSA_phys_, AH-(af)gHSA_4S_, AH-(af)gHSA_8S_, and AH-(af)gHSA_8US_ systems at excitation wavelength λ_ex_ = 275 nm than at λ_ex_ = 295 nm, probably indicates much easier drug access to tyrosyl residues (Tyrs) than to the tryptophanyl residue (Trp-214). Only for AH-(af)gHSA and AH-(af)gHSA_4US_ systems determined percentage differences in quenching fluorescence of albumin excited at λ_ex_ = 275 nm and λ_ex_ = 295 nm are within error limits of measurement, which indicates a greater participation of Trp-214 than Tyrs residues in the interaction. For the molar ratio of AH:albumin 10:1, a two-fold increase in the amount of saturated and unsaturated fatty acids compared to the physiological value of the acids causes (50.65% and 33.49% at λ_ex_ = 275 nm and 55.40% and 16.70% at λ_ex_ = 295 nm) a stronger quenching of the macromolecule fluorescence by AH.

Stern–Volmer K_SV_, biomolecular quenching rate k_q_ and association K_a_ constants determined from the Stern–Volmer, Klotz, and Hill methods, respectively, for AH complex with glycated, defatted albumin ((af)gHSA) are characterized by higher values than for AH complex glycated in the presence of fatty acids albumin (AH-(af)gHSA_phys_, AH-(af)gHSA_4S_, AH-(af)gHSA_8S_, AH-(af)gHSA_4US_, (af)gHSA_8US_), as previously discussed. The decrease in the association constant K_a_ value demonstrates the effect of fatty acids on the reduction of AH affinity towards human serum albumin. Similar conclusions were reached by Shum and Jusko [34], who showed a weakness of theophylline-albumin interaction with an increase in the concentration of fatty acids under the physiological concentration in the macromolecule. Based on the in vitro results regardin the effect of fatty acids on the binding of acetohexamide to glycated human serum albumin, it can be assumed that under conditions of abnormal fat content in the body, the pharmacokinetics of the drug may be affected. A four-fold increase in saturated and unsaturated fatty acids amount relative to the physiological value of acids increases the binding strength of AH to albumin, while an eight-fold increase in the amount of saturated and unsaturated fatty acids relative to the physiological value of acids reduces the binding strength of AH to albumin. Throughout a therapy with acetohexamide, it is important to control the amount of fatty acids supplied to the body with diet and/or in the form of supplements. Stronger binding of AH to albumin weakens its therapeutic effect, which affects the need to increase the dose of the drug in order to obtain normoglycemia, because, as it is known, only the free fraction of the drug has a therapeutic effect. However, the binding of AH with less strength to albumin increases its pharmacological activity, and thus the probability of side effects that can be dangerous to the patient′s health. The research suggests the need for individual dose selection, especially for an obese patient with a chronic disease, e.g., diabetes.

## 3. Materials and Methods

### 3.1. Reagents

Crystallized and lyophilized human serum albumin fatty acid free (fraction V) ((af)HSA, Lot No. 6312A) was purchased from MP Biomedicals SARL (Illkirch Cedex, France). Myristic acid (MYR, Lot No. R28576), oleic acid (OA, Lot No. 1912J), linoleic acid (LA, Lot No. 2353J), palmitic acid (PA, Lot No. 6798H) and stearic acid (SA, Lot No. 7729H) were provided by MP Biomedicals LLC (Irvine, CA, USA), Sodium azide (NaN_3_, Lot No. BCBD6941V), acetohexamide (AH, Lot No. SLBF5611V) were obtained by Sigma-Aldrich Chemical Co. (Darmstadt, Germany) and Sigma-Aldrich Chemical Co. (Shanghai, China), respectyvely. D( + )-glucose (GLC, Lot No. A0299881) was supplied by POCH S.A. (Gliwice, Poland). All chemicals were of the highest analytical quality. The stock solution of AH, MYR, OA, LA, PA, and SA were prepared by dissolving appropriate amounts in methanol from Merck KGaA (Darmstadt, Germany, Lot No. 32373611/18).

### 3.2. Procedure of Preparation Fatty Acids Solutions for Fluorescence and Absorbance Studies

Defatted human serum albumin ((af)HSA) in the presence of glucose (GLC) at 5 × 10^−6^ mol∙L^−1^ and 0.05 mol∙L^−1^ concentrations, respectively, were prepared in phosphate buffer solution (pH 7.4, 0.05 mol∙L^−1^) in double-distilled water and in the presence of sodium azide (NaN_3_) (0.015 mol∙L^−1^). Solution of protein was passed through a sterile Millex-GP syringe filters with 0.2 μm pores, and then it was incubated in sterile closed tubes for a period of 21 days at constant temperature of 37 °C. After the incubation period, to remove the excess of unbound glucose, the solutions of glycated human serum albumin (af)gHSA was dialyzed extensively against 0.05 mol∙L^−1^ phosphate buffer at pH 7.4 for 24 h.

To investigate the influence of fatty acids on glycated human serum albumin binding with acetohexamide (AH), the mixture of fatty acids (FA) corresponding to the physiological FA composition in human blood (mix 1) and FA four mixtures at different concentrations (mix 2–5) were prepared. Six glycated albumin samples were prepared without ((af)gHSA) and in the presence of a suitable fatty acid mixture ((af)gHSA_phys_, (af)gHSA_4S,_ (af)gHSA_8S,_ (af)gHSA_4US_, (af)gHSA_8US_). Fatty acids physiological mixture at 2 × 10^−5^ mol∙L^−1^ concentration (mix 1) contains FA mixtures at 8 × 10^−6^ mol∙L^−1^ (oleic acid, OA), 6 × 10^−6^ mol∙L^−1^ (palmitic acid, PA), 0.5 × 10^−6^ mol∙L^−1^ (stearic acid, SA), 0.5 × 10^−6^ mol∙L^−1^ (myristic acid, MYR) and 5 × 10^−6^ mol∙L^−1^ (linoleic acid, LA) concentrations. The next two mixtures contain four (mix 2) and eight times (mix 3) more saturated fatty acids (PA, MYR, SA) in relation to the physiological value. The total concentration of these solutions was 4.1 × 10^−5^ mol∙L^−1^ and 6.9 × 10^−5^ mol∙L^−1^. The last two solutions contain four (mix 4) and 8 times (mix 5) more unsaturated fatty acids (OA and LA) in relation to the physiological value. Total concentration of these solutions was 5.9 × 10^−5^ mol∙L^−1^ and 1.11 × 10^−4^ mol∙L^−1^. The stock solution of MYR, OA, LA, PA, and SA at 1 × 10^−3^ mol∙L^−1^ concentration was prepared by dissolving the right amount of acid in methanol, while mixtures of FA were prepared in phosphate buffer. A stock solution of acetohexamide (AH) at 5 × 10^−3^ mol∙L^−1^ concentration was prepared in methanol. The content of methanol in the samples did not exceed 1% of tested protein solution total volume.

### 3.3. Instruments and Measurements Conditions

The fluorescence and absorbance measurements of the samples were recorded at 37 °C using spectrofluorimeter JASCO FP-6500 (Hachioji, Tokyo, Japan) equipped with Peltier thermostat (∆t ± 0.2 °C) (error apparatus ± 1.5 nm) (Peltier thermostat is a part of JASCO FP-6500) and spectrophotometer JASCO V-760 (correcting error of apparatus for wavelength and photometric is equal to ± 0.3 nm and ± 0.002 Abs. at 0.5 Abs) (Hachioji, Tokyo, Japan), respectively. For the measurement standards quartz cuvettes 1 cm × 1 cm × 4 cm were used. The fluorescence spectra presented in the paper were corrected for the solvent dispersion (phosphate buffer) using the Spectra Manager program, and then analyzed using OriginPro version 8.5 SR1 software (Northampton, MA, USA). Finally, light scattering caused by buffer was subtracted from fluorescence of samples in each spectrum using software supplied by JASCO (Spectra Manager). The results of the study were expressed as a mean ± relative standard deviation (RSD) from three independent experiments. Linear regression by fitting experimental data to the appropriate equations were analyzed using OriginPro version 8.5 SR1 software (Northampton, MA, USA).

The analysis of the interaction of acetohexamide (AH) with glycated human serum albumin without (af)gHSA and in the presence of ((af)gHSA_phys_, (af)gHSA_4S,_ (af)gHSA_8S,_ (af)gHSA_4US_, (af)gHSA_8US_) fatty acids were done using albumin fluorescence quenching method. To excite fluorescence of the proteins, λ_ex_ = 275 nm (exites tyrosyls and tryptophanyl residues, λ_em_ = 285 nm − 400 nm) and λ_ex_ = 295 nm (excites tryptophanyl residue, λ_em_ = 305 nm − 400 nm) wavelengths were used. The measurements were conducted when the spectral width of the band (for monochromator of excitation and emission radiation) was equal to 3 nm, sample scanning speed of 200 nm/min, signal sensitivity “Medium”, response time 4 s. For measurements of drug-albumin and drug-albumin-FA mixtures (mix 1–5), solutions of proteins at constant 5 × 10^−6^ mol∙L^−1^ concentration were used, without (0) and in the presence of acetohexamide (AH) at final concentration: 5 × 10^−6^ mol∙L^−1^ (1), 1 × 10^−5^ mol∙L^−1^ (2), 1.5 × 10^−5^ mol∙L^−1^ (3), 2 × 10^−5^ mol∙L^−1^ (4), 2.5 × 10^−5^ mol∙L^−1^ (5), 3 × 10^−5^ mol∙L^−1^ (6), 3.5 × 10^−5^ mol∙L^−1^ (7), 4 × 10^−5^ mol∙L^−1^ (8), 4.5 × 10^−5^ mol∙L^−1^ (9), 5 × 10^−5^ mol∙L^−1^ (10). Samples for fluorescence measurements have been made by titration method. By the use of Hamilton syringe a suitable volume of titrant (3 μL in 10 portions) has been added to 3 mL of albumins immediately before the fluorescence measurement. The final AH:(af)gHSA, AH:(af)gHSA_phys_, AH:(af)gHSA_4S_, AH:(af)gHSA_8S_, AH:(af)gHSA_4US_ and AH:(af)gHSA_8US_ molar ratio was 10:1. The degree of glycated albumin fluorescence quenching by the acetohexamide was determined relative to the fluorescence of the non-ligand albumin solutions. Due to the inner filter effect (IFE) caused by the presence of the drug, the recorded fluorescence was corrected using the following formula [20]:

(1) $$F_{cor}=F_{obs}\cdot 10^{\left(\frac{A_{ex}+A_{em}}{2}\right)}$$

where: $F_{cor}$ and $F_{obs}$ are corrected and observed fluorescence intensity, respectively; $A_{ex}$ and $A_{em}$ are the absorbance at excitation (λ_ex_ = 275 nm or λ_ex_ = 295 nm) and emission wavelength for (af)gHSA (λ_ex_ = 275 nm: λ_em_ = 327- 323 nm or λ_ex_ = 295 nm: λ_em_ = 334 nm) and (af)gHSA_phys_, (af)gHSA_4S,_ (af)gHSA_8S,_ (af)gHSA_4US_, (af)gHSA_8US_ (λ_ex_ = 275 nm: λ_em_ = 323 nm or λ_ex_ = 295 nm: λ_em_ = 329–332 nm), respectively.

### 3.4. The Analyzis of Acetohexamide-Albumins Interaction

Based on the calculated fluorescence emission intensities without and in the presence of fatty acids glycated human serum albumin, the quenching curves ($\frac{F}{F_{0}}$ vs. ligand:albumin molar ratio, where: F and $F_{0}$ is the fluorescence intensity at the maximum wavelength of albumin in the presence and absence of a quencher, respectively) of (af)gHSA, (af)gHSA_phys_, (af)gHSA_4S,_ (af)gHSA_8S,_ (af)gHSA_4US_ and (af)gHSA_8US_ in the presence of acetohexamide (AH) have been plotted.

The course of Stern–Volmer curves (Equation (2)) allowed to conclude the mechanism of quenching fluorescence of albumins fluorophores by the studied drug [27].

(2) $$\frac{F_{0}}{F}=1+k_{q}\tau_{0}\cdot \left[L\right]=1+K_{SV}\cdot \left[L\right]$$

where: $k_{q}$ is the bimolecular quenching rate constant [mol^−1^∙L∙s^−1^]; $\tau_{0}$ is the average fluorescence lifetime of albumin without of quencher $\tau_{0}$ = 6.0 × 10^−9^ s [30]; $K_{SV}$ is the Stern–Volmer constant [mol^−1^∙L]; [L] is the ligand concentration [mol∙L^−1^]; $\left[L\right]=\left[L_{b}\right]+[L_{f}]$, $\left[L_{b}\right]$ and $\left[L_{f}\right]$ are the bound and free (unbound) drug concentrations [mol∙L^−1^].

From the modified Stern–Volmer relationship (Equation (3)), the Stern–Volmer K_SV_ constant in AH:(af)gHSA, AH:(af)gHSA_phys_, AH:(af)gHSA_4S_, AH:(af)gHSA_8S_, AH:(af)gHSA_4US_, AH:(af)gHSA_8US_ systems and also the fractional maximum protein fluorescence accessible for the quencher (f_a_) were determined [29].

(3) $$\frac{F_{0}}{\Delta F}=\frac{1}{\left[L\right]}\cdot \frac{1}{f_{a}}\cdot \frac{1}{K_{SV}}+\frac{1}{f_{a}}$$

where: $f_{a}$ is the fractional maximum protein fluorescence accessible for the quencher.

Isotherms of drug binding to without and in the presence of fatty acids glycated human serum albumin have been obtained based on the graph of the function $r=f\left(\left[L_{f}\right]\right)$, where: $r=\frac{\left[L_{b}\right]}{\left[\left(af\right)gHSA\right]}$ is the number of ligands moles bound per mole of protein molecule; $\left[L_{b}\right]=\frac{\Delta F}{\Delta F_{max}}\cdot \left(af\right)gHSA_{total}$, ΔF is the difference between $F_{0}$ and F, $\Delta F_{max}$ (maximal fluorescence change with complete saturation) is evaluated from the linear part of the $\frac{1}{\Delta F}$ vs. $\frac{1}{\left[L\right]}$; [(af)gHSA] is glycated serum albumin concentration [mol∙L^−1^] [23].

From the Klotz curves (Equation (4)) [35], in addition to the values of K_a_ association constants, the number of drug molecules bound to one protein molecule (*n*) was determined.

(4) $$\frac{1}{r}=\frac{1}{n}+\;\frac{1}{n\cdot K_{a}\cdot [L_{f}]}$$

where: n is the number of binding sites for the independent class of drug binding sites in the glycated albumin molecule.

From Hill dependence (Equation (5)) [36] association constants K_a_ and interaction coefficients $n_{H}$ were calculated.

(5) $$log\left(\frac{r}{1-r}\right)=n_{H}\cdot log[L_{f}]+logK_{a}$$

where: $n_{H}$ is the Hill’s coefficient.

### 3.5. Binding Sites Visualisation. Molecular Docking Simulation

To visualize the potential binding site of acetohexamide (AH) to glucose complexed human serum albumin (GLC-HSA), molecular docking simulation has been made using the CLC Drug Discovery Workbench ver. 1.0.2. (CLC Bio, a QIAGEN Company: Aarhus, Denmark) [License number: CLC-LICENSE-51JT8-DXYBY-2A3EW-ED80P-DGW80]. The crystallographic structure of glucose complexed human serum albumin (GLC-HSA) has been obtained from the PDB protein structure database (Protein Data Bank) using the PDB ID: 4IW2 [37]. Theoretical structure of atetohexamide (AH) was minimized and optimized by the Austin Model 1 (AM1)-semi-empirical method using the Atomic and Molecular Electronic Structure System (GAMESS) [38]. The AH docking procedure for the GLC-HSA structure was conducted simulating physiological conditions, in accordance with the default program configuration.

## 4. Conclusions

Fluorescence spectroscopy is a technique that allows to monitor the intermolecular interactions. To determine the distribution capacity and binding properties of glycated, defatted albumin ((af)gHSA) and albumin containing a physiological mixture of fatty acids ((af)gHSA_phys_), albumin containing 4 times ((af)gHSA_4S_) and 8 times ((af)gHSA_8S_) higher amount of saturated fatty acids (FA_S_) and 4 times ((af)gHSA_4US_) and 8 times ((af)gHSA_8US_) higher amount of unsaturated (FA_US_) fatty acids in relation to physiological concentration, a number of fluorescent quenching measurements of albumin fluorescence by acetohexamide (AH) were made. Based on the quantitative and qualitative analysis we can conclude that the presence of both saturated and unsaturated fatty acids disturbs the binding of acetohexamide to human serum albumin. Acetohexamide binds more strongly to defatted albumin (af)gHSA than to albumin in the presence of fatty acids. Competitive binding of AH and fatty acids to albumin may influence the concentration of free drug fraction and thus its therapeutic effect. It should be taken into account that under conditions of abnormal fat content in the body, the pharmacokinetics of the drug may be affected.

## Figures and Tables

**Table 1:** Stern-Volmer constants K_SV_ (mol^−1^∙L), biomolecular quenching rate constants k_q_ (mol^−1^∙L∙s^−1^) and maximum available fluorescence fraction of all albumin fluorophores f_a_ calculated for the AH-(af)gHSA and AH-(af)gHSA_phys_ systems; λ_ex_ = 275 nm and λ_ex_ = 295 nm.

**λ_ex_ = 275 nm**	K_SV_ ± RSD ^*)^ × 10^4^ (mol^−1^∙L)	^a^ k_q_ ± RSD ^*)^ ** × 10^12^ (mol^−1^∙L∙s^−1^)**	f_a_ ± RSD ^*)^
AH-(af)gHSA	4.11 ± 0.04	6.85 ± 0.06	0.43 ± 0.01
AH-(af)gHSA_phys_	0.23 ± 0.01	0.38 ± 0.02	1.00 ± 0.01
**λ_ex_ = 295 nm**	K_SV_ ± RSD ^*)^ × 10^4^ (mol^−1^∙L)	^a^ k_q_ ± RSD ^*)^ ** × 10^12^ (mol^−1^∙L∙s^−1^)**	f_a_ ± RSD ^*)^
AH-(af)gHSA	3.10 ± 0.05	5.17 ± 0.08	0.50 ± 0.01
AH-(af)gHSA_phys_	0.10 ± 0.01	0.17 ± 0.02	1.00 ± 0.01

^*)^ relative standard deviation; **^a^** calculated using: $k_{q}=\frac{K_{\mathrm{SV}}}{\tau_{0}}$, where: $\tau_{0}$ = 6.0 × 10^−9^ s [30]—the average fluorescence lifetime of albumin without quencher.

**Table 2:** Association constants K_a_ (mol^−1^∙L), mean number of AH moles bound with one mole of (af)gHSA and (af)gHSA_phys_ (*n*), the Hill coefficient (n_H_) in AH-(af)gHSA and AH-(af)gHSA_phys_ system; λ_ex_ = 275 nm, λ_ex_ = 295 nm.

	Klotz Method		Hill Method	
**λ_ex_ = 275 nm**	K_a_ ± RSD ^*)^ ** × 10^4^ (mol^−1^∙L)**	*n* ± RSD ^*)^	K_a_ ± RSD ^*)^ ** × 10^4^ (mol^−1^∙L)**	n_H_ ± RSD ^*)^
AH-(af)gHSA	5.42 ± 0.06	0.93 ± 0.02	4.64 ± 0.01	0.98 ± 0.02
AH-(af)gHSA_phys_	0.50 ± 0.17	1.00 ± 0.43	0.43 ± 0.02	0.95 ± 0.02
**λ_ex_ = 295 nm**	K_a_ ± RSD ^*)^ ** × 10^4^ (mol^−1^∙L)**	*n* ± RSD ^*)^	K_a_ ± RSD ^*)^ ** × 10^4^ (mol^−1^∙L)**	n_H_ ± RSD ^*)^
AH-(af)gHSA	3.87 ± 0.07	0.93 ± 0.03	3.45 ± 0.03	1.00 ± 0.02
AH-(af)gHSA_phys_	0.74 ± 0.20	0.96 ± 0.32	1.52 ± 0.02	1.27 ± 0.01

^*)^ relative standard deviation.

**Table 3:** Stern-Volmer constants K_SV_ (mol^−1^∙L), biomolecular quenching rate constants k_q_ (mol^−1^∙L∙s^−1^) and the fraction of maximum available fluorescence of albumin f_a_ calculated for the AH-(af)gHSA_4S_, AH-(af)gHSA_8S_, AH-(af)gHSA_4US_ and AH-(af)gHSA_8US_ system; λ_ex_ = 275 nm and λ_ex_ = 295 nm.

**λ_ex_ = 275 nm**	K_SV_ ± RSD ^*)^ × 10^4^ (mol^−1^∙L)	^a^ k_q_ ± RSD ^*)^ ** × 10^11^ mol^−1^∙L∙s^−1^)**	f_a_ ± RSD ^*)^
AH-(af)gHSA_4S_	0.83 ± 0.19	13.83 ± 3.17	0.13 ± 0.04
AH-(af)gHSA_8S_	0.49 ± 0.01	8.17 ± 0.17	1.00 ± 0.01
AH-(af)gHSA_4US_	0.08 ± 0.01	1.33 ± 0.01	1.00 ± 0.01
AH-(af)gHSA_8US_	0.14 ± 0.01	2.33 ± 0.01	1.00 ± 0.01
**λ_ex_ = 295 nm**	K_SV_ ± RSD ^*)^ × 10^4^ mol^−1^∙L)	^a^ k_q_ ± RSD ^*)^ ** × 10^11^ (mol^−1^∙L∙s^−1^)**	f_a_ ± RSD ^*)^
AH-(af)gHSA_4S_	0.77 ± 0.06	12.83 ± 1.00	0.08 ± 0.01
AH-(af)gHSA_8S_	0.25 ± 0.01	4.17 ± 0.17	1.00 ± 0.01
AH-(af)gHSA_4US_	0.09 ± 0.01	1.50 ± 0.01	1.00 ± 0.01
AH-(af)gHSA_8US_	0.11 ± 0.01	1.83 ± 0.01	1.00 ± 0.01

^*)^ relative standard deviation; **^a^** calculated using: $k_{q}=\frac{K_{\mathrm{SV}}}{\tau_{0}}$, where: $\tau_{0}$ = 6.0 × 10^−9^ s [30]—the average fluorescence lifetime of albumin without quencher.

**Table 4:** Association constants K_a_ (mol^−1^∙L), mean number of AH moles bound with one mole of (af)gHSA_4S_, (af)gHSA_8S_, (af)gHSA_4US_ and (af)gHSA_8US_ (*n*), the Hill coefficient (n_H_) in AH-(af)gHSA_4S_, AH-(af)gHSA_8S_, AH-(af)gHSA_4US_ and AH-(af)gHSA_8US_ system; λ_ex_ = 275 nm and λ_ex_ = 295 nm.

	Klotz Method		Hill Method	
**λ_ex_ = 275 nm**	K_a_ ± RSD ^*)^ ** × 10^4^ (mol^−1^∙L)**	*n* ± RSD ^*)^	K_a_ ± RSD ^*)^ **10^4^ (mol^−1^∙L)**	n_H_ ± RSD ^*)^
AH-(af)gHSA_4S_	0.92 ± 0.20	0.93 ± 0.25	-*	-*
AH-(af)gHSA_8S_	0.39 ± 0.07	0.98 ± 0.19	0.37 ± 0.01	0.99 ± 0.01
AH-(af)gHSA_4US_	0.70 ± 0.09	0.97 ± 0.07	0.68 ± 0.01	1.00 ± 0.01
AH-(af)gHSA_8US_	0.23 ± 0.05	0.99 ± 0.05	0.27 ± 0.02	1.02 ± 0.02
**λ_ex_ = 295 nm**	K_a_ ± RSD ^*)^ 10^4^ (mol^−1^∙L)	*n* ± RSD ^*)^	K_a_ ± RSD ^*)^ ** × 10^4^ (mol^−1^∙L)**	n_H_ ± RSD ^*)^
AH-(af)gHSA_4S_	0.85 ± 0.06	0.94 ± 0.08	-*	-*
AH-(af)gHSA_8S_	0.52 ± 0.04	0.98 ± 0.09	0.51 ± 0.01	1.00 ± 0.01
AH-(af)gHSA_4US_	0.87 ± 0.08	0.95 ± 0.05	2.09 ± 0.01	1.42 ± 0.04
AH-(af)gHSA_8US_	0.67 ± 0.06	0.96 ± 0.08	1.37 ± 0.02	1.28 ± 0.02

^*)^ relative standard deviation; -*—unable to calculate.
